# Is L‐Arginine an Appropriate Alternative for Conventional Anti‐Atherosclerotic Therapy?: A Comprehensive Review

**DOI:** 10.1002/hsr2.70544

**Published:** 2025-03-27

**Authors:** Ali Mortezaei, Mohammad Ghorbani, Bardia Hajikarimloo, Omar Sameer, Toba Kazemi, Ebrahim Salavati, Mohsen Hamidpour, Mohammad Esmail Gheydari

**Affiliations:** ^1^ Student Research Committee Gonabad University of Medical Sciences Gonabad Iran; ^2^ Faculty of Allied Medicine, Department of Medical Laboratory Sciences Gonabad University of Medical Sciences Gonabad Iran; ^3^ Department of Hematology and Blood Banking, School of Allied Medical Sciences Shahid Beheshti University of Medical Sciences Tehran Iran; ^4^ School of Medicine Shahid Beheshti University of Medical Sciences Tehran Iran; ^5^ College of Medicine University of Sharjah Sharjah UAE; ^6^ Cardiovascular Diseases Research Center Birjand University of Medical Sciences Birjand Iran; ^7^ Allameh Bohlool Hospital Gonabad University of Medical Sciences Gonabad Iran; ^8^ HSC Research Center, Department of Hematology and Blood Banking, School of Allied Medical Sciences Shahid Beheshti University of Medical Sciences Tehran Iran; ^9^ Department of Cardiology, School of Medicine, Taleghani Hospital Shahid Beheshti University of Medical Sciences Tehran Iran

**Keywords:** cardiology, neurology, pathology and laboratory medicine

## Abstract

**Background:**

Atherosclerosis is the leading cause of cardiovascular disease (CVD). Historically, the management of atherosclerosis was focused on decreasing lipid profile levels; however, recent evidence demonstrated that platelets and leukocytes play an important role in forming and exacerbating atherosclerosis. L‐arginine (L‐Arg), a precursor to nitric oxide (NO), plays a critical role in modulating oxidative stress and influencing platelet‐leukocyte recruitment and has been extensively addressed in the context of CVD.

**Objective:**

We aimed to perform a comprehensive literature review on l‐Arg metabolism in the causative pathway of atherosclerosis compared to conventional treatment and it as a putative therapeutic approach.

**Results:**

L‐Arg supplementation has shown promising effects on NO production, improving endothelial function and reducing oxidative stress in preclinical models. Clinical studies have indicated moderate improvements in vascular health markers, including reductions in inflammation and oxidative stress, although results have varied across studies. The potential of l‐Arg to modify platelet‐leukocyte recruitment and slow the progression of atherosclerotic plaque development has been observed in certain studies. However, these benefits remain inconsistent, and more robust clinical trials are needed to confirm its effectiveness. Additionally, while l‐Arg appears to be relatively safe, some studies reported mild gastrointestinal discomfort as a common side effect.

**Conclusion:**

l‐Arg holds potential as a complementary or alternative treatment for atherosclerosis, particularly in improving endothelial function and reducing inflammation and oxidative stress. However, the variability in clinical outcomes and the lack of long‐term data required further investigation into assessing therapeutic benefits. Future studies should focus on determining optimal dosing regimens, evaluating their long‐term safety, and assessing their potential in combination with other therapies to enhance cardiovascular outcomes.

## 1 Introduction

Cardiovascular disease (CVD) is a significant cause of death that takes more lives annually than any other disease with atherosclerosis being the main causative pathology [1]. CVD causes virtually one out of every three fatalities in the United States [1], as well as 45% of female and 39% of male fatalities in Europe [2]. By 2030, CVD is anticipated to be responsible for 23.6 million people's death globally annually [1, 2]. Also, the prevalence of CVD increases with age in both males and females, although it is higher in men [1]. Approximately 47% of all Americans have at least one of the three major CVD risk factors, which include high blood pressure, hyperlipidemia, and smoking [3]. Previous evidence demonstrated atherosclerosis as a lipoprotein‐driven and chronic inflammatory disease, hence most of approved therapies focus on low‐density lipoprotein (LDL) cholesterol lowering [4]. Recent understanding of pathogenesis of atherosclerosis explained three stages as imbalanced lipid metabolism integrated with misconducted immune response leading to chronic inflammatory process in the blood vessel wall. For many years, platelets were known as a key player in thrombosis and hemostasis, but recent studies demonstrate the causative relationship between atherosclerosis and the three major bases: cellular effects, signaling, and inflammation [5, 6]. Novel investigations showed a complex interplay of leukocytes and platelets with specific receptors adhering to the endothelial layer of the vessels, which may serve as one of the main causes of atherosclerosis. These circumstances lead to platelet‐leukocyte aggregates (PLA) formation, which is exacerbated by stress, thrombosis, lipid accumulation, endothelial damage, immunological response, and activation of platelets or leukocytes [4, 5, 6].

Recent experimental studies suggest that platelet‐leukocyte recruitment instigate the growth and expansion of atherosclerosis plaque by increasing leukocyte phagocytic activity, production of reactive oxygen species (ROS), leukocyte transmigration over the vascular endothelial inner layer, activation of coagulation via tissue factor, Netosis and neuroendocrine tumor [7, 8, 9]. In this regard, the Canakinumab Anti‐inflammatory Thrombosis Outcomes Study demonstrated that anti‐inflammatory therapy targeted interleukin‐1β independently of lipid‐lowering drugs, significantly reduced high‐sensitivity C‐reactive protein and the incidence of recurrent CVD [10]. An in vivo study showed anti‐inflammatory drugs such as hydroxyurea are effective for regressing the plaques more than conventional antithrombotic, and they also demonstrated combining them with statin and antiplatelet roughly eliminates the atherosclerosis plaque [11]. Another study utilizing methotrexate in a Rabbit Model of in‐stent neoatherosclerosis exhibited a reduction in plaque formation and long‐term risk of late thrombosis [12]. The regression of the plaque by the drugs mentioned above is due to the decrease in the recruitment of white blood cells (WBCs) in endothelial cells. Nevertheless, these medications require special consideration for their complications and toxicity. Thereby, there is a need for alternative therapeutics that are low‐cost, available, non‐invasive, have a lower risk of recurrence and favorable outcomes.

Cellular factors interactions, such as endothelial cells, platelets, and WBC, with cellular mediators and biochemical factors, may have contributed to the progression and acceleration of plaque formation, which certain amino acids and their metabolism may affect them. l‐arginine (l‐Arg) is a semi‐essential amino acid that has two metabolism pathways and serves as a precursor of nitric oxide (NO) that affects oxidative stress, platelet‐leukocyte recruitment, and oxidative stress has been extensively addressed in the context of CVD. The current study focuses on l‐Arg metabolism in the causative pathway of atherosclerosis compared to conventional treatment and it as a putative therapeutic approach in atherosclerosis.

## 2 Inhibition of Cellular Factors

### 2.1 Endothelial Cells

**Figure 1:**
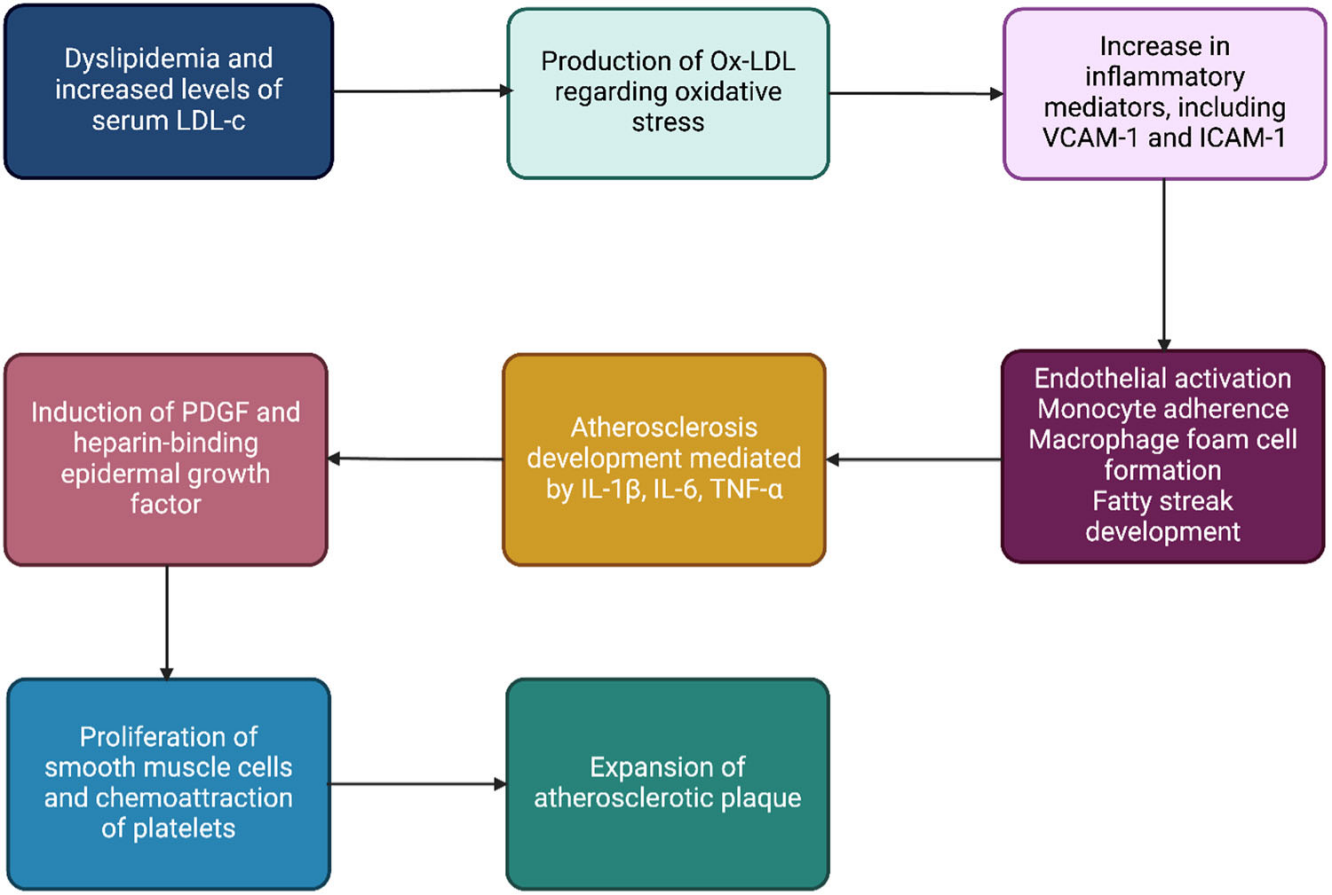
Molecular process of the atherosclerosis plaque formation.

**Figure 2:**
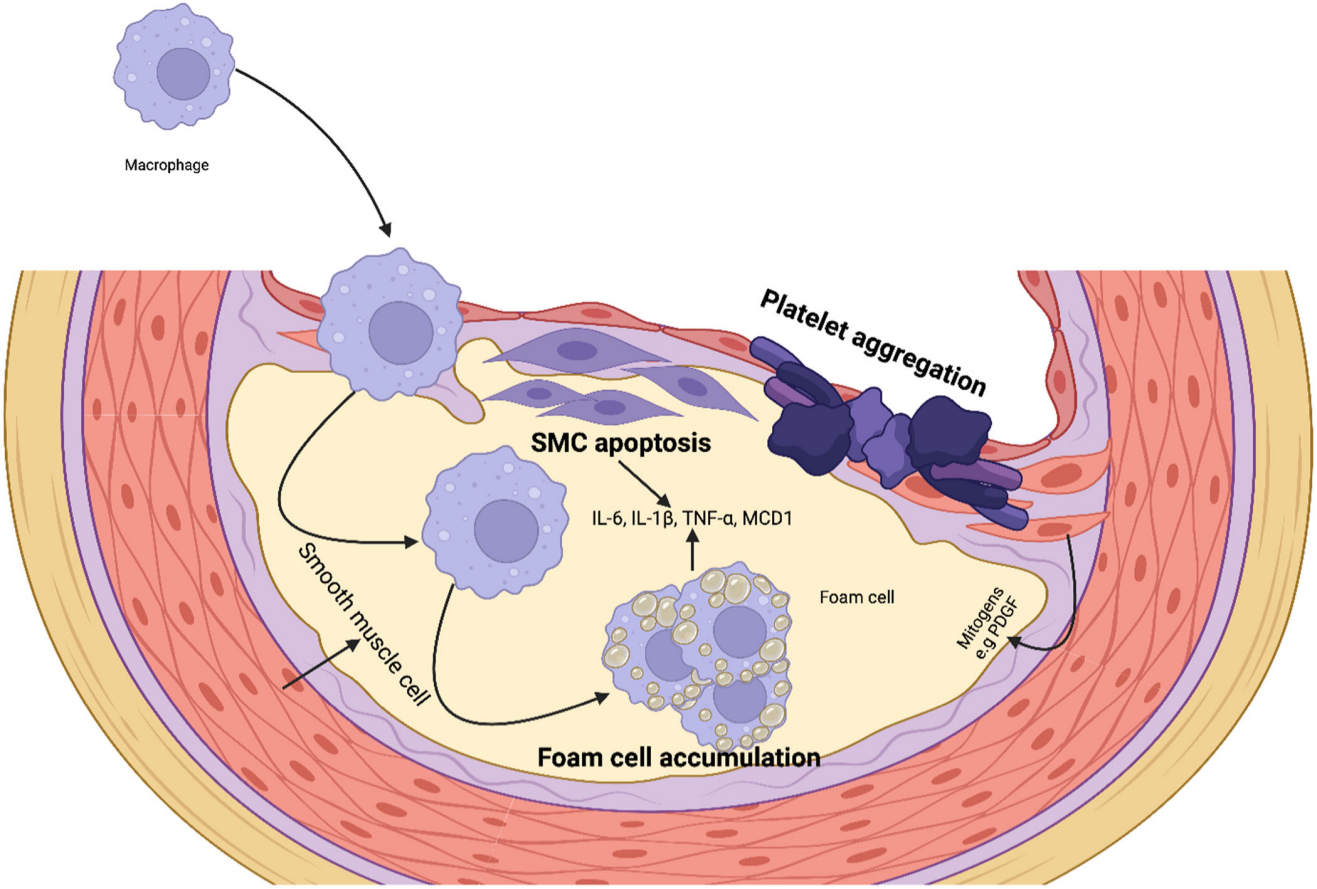
Leukocytes and smooth muscle cell interaction in plaque formation.

Endothelium provides the basis of the atheroma formation and its interface between the blood cells [13, 14]. Endothelial cells are pivotal in angiogenesis, and their injury or dysfunction represents a key factor in the progression of CVDs [15, 16]. Research indicates that after vascular graft implantation, inadequate endothelialization coupled with excessive SMCs proliferation over time can contribute to developing of thrombosis [15, 16]. High‐cholesterol diet can alter the expression of vascular cell adhesion protein and other adhesion molecules that result in an increased bind of leukocytes to the endothelium. On the other hand, this can also enhance the attachment of chemoattractants that facilitate the passage of leukocytes into the intima [13, 14]. Endothelial cells expressing endothelial nitric oxide synthesis (eNOS) lead to hydrolyze l‐Arg into NO, which has vasodilation effects and ameliorates atheroprotective functions Figures 1 and 2 [4]. In atherosclerosis, eNOS may become uncoupled, producing ROS rather than NO, resulting in endothelial dysfunction. Whereas l‐Arg is metabolized by arginase, l‐homoarginine serves as an arginase regulator [4]. Furthermore, some conditions such as oxidative stress, LDL, and cholesterol, as well as limited bioavailability of l‐Arg, eNOS, and NO can alter the l‐Arg hydrolyze pathway and produce uncoupled eNOS. This vicious cycle produces superoxide instead of NO, which oxidizes LDL and promotes endothelial cell dysfunction [17, 18]. Correspondingly, Kuhlencordt et al. [19] studied ApoE‐/‐ knockout mice with eNOS deficiency, which had more evidence of hypertension and atherosclerosis risk. On contrary, Shi et al. [20] using eNOS‐deficient mice showed paradoxically the absence of eNOS‐mediated LDL oxidation may diminish the risk of atherosclerosis. l‐Arg activates eNOS, leading to NO synthesis, which plays a critical role in modulating vascular tone, regulating thrombosis formation, and mediating inflammatory processes [21, 22]. Recent research has shown that l‐Arg, when used as an adjunct to budesonide, enhances NOS expression in endothelial cells, providing a substrate for eNOS and facilitating NO synthesis [22]. Furthermore, in vivo treatment with budesonide and l‐Arg loaded nanoparticles can significantly reduce the extent of arteriosclerotic plaque formation [22, 23]. Moreover, l‐Arg serves as a substrate for endothelial cells, which release extracellular vesicles containing NOS enzymes and bioactive molecules as well as NO [24]. These components play a crucial role in maintaining vascular homeostasis, further highlighting l‐Arg's contribution to atherosclerosis [24].

Proteomic analysis revealed that the physiological level of l‐Arg (e.g., 0.1 mM) affects the expression of structural proteins (vimentin and tropomyosin) and cytochrome bc1 in coronary venular endothelial cells, which also had beneficial effects on the cellular redox state and enhanced NO production in endothelial cells [25]. A study evaluated the effect of l‐Arg on the circulatory system in a rabbit model of hypercholesterolemia. The finding indicated that l‐Arg supplementation statistically significantly affects endothelial progenitor cells, angiogenesis, NO, decreased ICAM1 and VCAM in endothelial cells, and prevented von Willebrand Factor increment and atherosclerosis development [26].

### 2.2 Platelets

**Figure 3:**
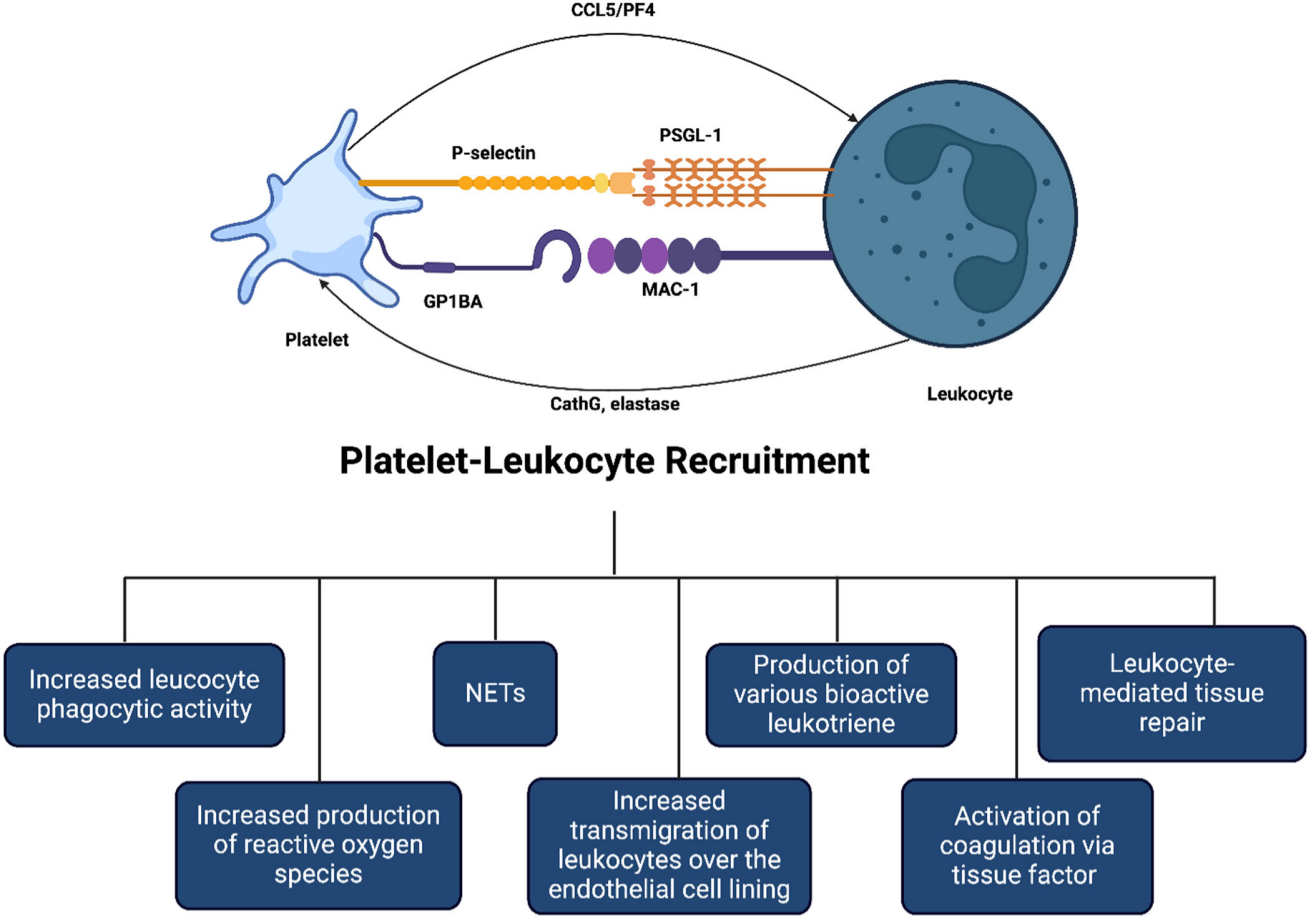
Platelet‐leukocyte aggregation mechanism in atherosclerosis.

As an inflammatory mediator, platelets interact with ECs and leukocytes (mainly monocytes) to participate in the formation of atherosclerotic lesions. Pro‐atherosclerosis factors can easily lead to platelet activation. The activated platelets express a variety of adhesion receptors. These receptors bind their matrix proteins (GPIb‐vWF, GPVI‐collagen, and GPIIb‐IIIa‐fibrinogen) to mediate platelet adhesion on ECs or leukocytes (P‐selectin glycoprotein ligand‐1 [PSGL] and Clec2‐PDPN) [27]. Moreover, platelet activation releases a variety of inflammatory factors (cytokines, chemokines, growth factors, and others). These inflammatory mediators can also be induced and expressed in neighboring cells, such as monocytes/macrophages, neutrophils, and ECs, which in turn affected platelets. Also, activated platelets by binding to WBCs and forming platelet leukocyte aggregate. Lead to recruitment of WBCs, especially monocytes and neutrophils to plaque and the development of atherosclerotic plaque [27]. l‐Arg scavenges aggregation, augments cyclic GMP levels, and promotes the Soluble guanylyl cyclase in collagen‐activated platelets [28]. A prospective, double‐blind, randomized crossover trial evaluated the 12 healthy men who received oral l‐Arg (7 g/oral day) and showed that l‐Arg inhibited platelet aggregation through the NO pathway [29]. Studies revealed that dysfunctional vascular endothelium, thinning of endothelial glycocalyx, functional alteration induced by impaired NO bioavailability, and elevated level of von Willebrand factor contribute to platelet activation, which l‐Arg has been shown can exert inhibition effects on those factors and furnish stable hemodynamics [30, 31, 32]. The known functions of platelet‐derived NO are shown in Figures 1 and 3. l‐Arg can limit the main functions of platelets in thrombosis formation such as, adhesion, aggregation, and degranulation through the production of NO.

### 2.3 Leukocytes Recruitment

Atherosclerosis is the leading cause of death worldwide and leukocyte recruitment is a key element of this phenomenon, thus allowing immune cells to enter the arterial wall. There, in concert with accumulating lipids, the invading leukocytes trigger a plethora of inflammatory responses which promote the influx of additional leukocytes and lead to the continued growth of atherosclerotic plaques. The recruitment process follows a precise scheme of tethering, rolling, firm arrest, crawling, and transmigration and involves multiple cellular and subcellular players. Circulating leukocytes are initially captured and begin rolling along the endothelium layer to commence the leukocyte recruitment cascade Figure 1 [33]. Furthermore, macrophage capping protein 1, a protein that modulates actin dynamics, plays an important role in macrophage migration and inflammatory responses, which are critical in atherosclerosis plaque formation and instability [34] (Figure 1). Intercellular adhesion molecule 1 (ICAM‐1) and other endothelial ligands are bound by activated 2 integrins like LFA‐1 (CD11a/CD18) and Mac‐1 (CD11b/CD18), resulting in firm adhesion to the inflamed endothelium cells and transmigration through a chemokine gradient [33, 35]. Polymorphonuclear leukocytes (PMNs) catabolize the l‐Arg by expressing Arginase‐1 and iNOS; subsequently, this depletion may locally suppress T‐cell functions [35]. Similarly, PMN arginase mitigating the l‐Arg levels suppressed the human natural killer [36]. Furthermore, l‐Arg reduces leukocyte recruitment [37] through the inhibition of ICAM‐1 on endothelial cells [38] and induces anti‐inflammatory effects on the PMNs. Also, studies showed that l‐Arg mitigated the ex vivo leukocyte recruitment in term‐born infants, whereas no statistically significant effect was distinct in preterm‐born infants [39]. Moreover, regarding the protective role of l‐Arg in CVD, it modulates the proteins involved in the cellular redox system, remodeling of extracellular matrix, and inflammatory activation of aortic interstitial valve cells, which leads to preventing osteogenic differentiation of aortic valve cells and reduces matrix calcification [40]. Aortic valve cell calcification may provide the substrate for leukocyte recruitment and highlight the production role of l‐Arg as scavenging vasoconstriction and leukocyte recruitment. Knight et al. [41] evaluated the apolipoprotein E‐deficient (ApoE−/−) mice revealed that l‐Arg reduced the neutrophil and macrophage recruitment, and delayed time to carotid artery thrombosis. Mitochondrial dysfunction is closely linked to the progression of atherosclerotic plaques. Studies indicate that increased mitochondrial ROS production is associated with plaque development, as observed in hypercholesterolemic LDL receptor‐deficient (LDLR−/−) and ApoE−/− mouse models [42, 43, 44]. Similarly, human studies have demonstrated that reduced mitochondrial oxygen consumption contributes to the formation of fibrous caps and necrotic core regions, further highlighting the role of mitochondrial dysfunction in plaque progression [45].

### 2.4 Smooth Muscle Cells Proliferation

The proliferation of vascular smooth muscle cells (VSMCs) under pathological conditions directly contributes to the progression of CVDs, such as atherosclerosis, hypertension, and restenosis. Native LDL as a mitogenic molecule in endothelial dysfunction lesions is a major independent risk factor for the progression of VSMCs and atherosclerosis [46, 47, 48]. However, long‐term oral l‐Arg (3 g/d) in 133 patients with peripheral arterial disease did not enhance the NO synthesis and vascular reactivity [49]. Evidence showed that increased arginase activity provoked the formation of spermine, which may induce mitochondrial Ca2+ absorption through mitochondrial p32 protein and put the mitochondria in a pathological posture to stimulate ROS generation, cytochrome C release, apoptosis, and agonist‐induced vasoconstriction activity [50, 51, 52]. Platelet‐derived growth factor and heparin‐binding epidermal growth factor are pivotal in the pathophysiology of CVD [53]. They drive smooth muscle cell (SMC) proliferation and migration, which contribute to vascular remodeling, the formation of intimal hyperplasia, and the activation of endothelial cells, thereby exacerbating the progression of atherosclerosis [53] (Figure 1).

Limonin as an arginase inhibitor prevented phosphorylation of PKCβII by native low‐density lipoprotein (nLDL) stimulation via a decrease in mitochondrial ROS formation. Therefore, arginase inhibitor, limonin, showed antiproliferative effect in nLDL‐stimulated VSMC. that increased intracellular l‐Arg by arginase inhibition had beneficial effects on the prevention of VSMC proliferation in the presence of high concentrations of nLDL. However, it is still unclear whether l‐Arg supplementation may have the same effect. Moreover, increased intracellular l‐Arg by arginase prevention had beneficial effects on the inhibition of VSMCs proliferation in the presence of high concentrations of native LDL [52]. Similarly, another study showed elevated l‐Arg levels after inhibiting limonin‐dependent arginase hampered native LDL‐stimulated VSMC proliferation [46]. Although, whether l‐Arg supplementation may have the same effect remains elusive.

### 2.5 PLA

Platelets are well known for their role in thrombotic disease, and traditionally, antiplatelets and anticoagulants are constantly utilized in the treatment and prevention of myocardial infarction and stroke [54]. Recent studies demonstrated that platelets function exceeds the thrombosis formation and plays a role in inflammation, facilitating tissue repair and expansion of various cancer metastasis [55], which the majority of them exerted in contributing to interactions between platelets and other circulating cell types [56, 57]. Leukocyte's attraction to the developing atherosclerotic plaque triggers atherogenesis and subsequently, platelets facilitate the leukocyte adhesion to the endothelial cells and transmigration through the layers [58]. Furthermore, platelets induce the formation of foam cells from adherent macrophages [54]. PLA participates in CVD. Increased levels of PLA were revealed in acute and chronic coronary syndromes, carotid stenosis, cardiovascular risk factors, and platelets by binding to WBCs and forming platelet leukocyte aggregate, causing functional, content, and morphological changes in WBCs. These changes are shown in Figure 3. Also, l‐Arg generates the NO in the presence of high Ca2+ levels, which may prevent the PLA [51, 52, 54]. Literature elucidated the momentous effect of PLA, particularly monocytes, on atherogenesis and CVD.

Studies demonstrated the predictive role of neutrophil counts in future coronary events [59, 60], and other evidence showed that local neutrophil accumulation is related to outcomes of CVD [61]. Murine models have shown that neutrophil extracellular traps (NET) primed macrophages for interleukin‐1β secretion, activating T helper 17 cells that reinforce leukocyte recruitment in atherosclerotic plaque formation, prompt oxidative stress, and oxidize high‐density lipoprotein particles [62, 63], while inhibition of factors required for NET formation mitigated the size of the plaque [41]. Moreover, NET promotes endothelial cell dysfunction and apoptosis and induces the production of anti‐double‐stranded‐DNA autoantibodies. Animal studies showed that l‐Arg by inhibiting NET formation can reduced atherosclerosis and arterial thrombosis in ApoE**–**/**–** murine [41]. Notably, NET furnishes a previously unsuspected association between prothrombotic molecules accumulation, such as von Willebrand factor and fibrinogen, and subsequently significantly contributing to plaque formation [64]. l‐Arg by generating NO when the platelet NO production and responsiveness are suppressed in hypertension, could prevent the monocyte‐platelet aggregates [65]. In summary, by producing NO in platelets, arginine reduces p‐selectin on the surface of platelets. Further, with the reduction of p‐selectin, its binding to PSGL on the surface of WBCs is reduced, and therefore, the formation of PLA is reduced, and as a result, the recruitment of WBCs to the atherosclerotic plaque is also reduced.

## 3 Inhibition of Cytokine and ROS

### 3.1 Production ROS

ROS possess a meaningful role in preserving the vascular proper condition, but to preclude inflammation and endothelial dysfunction, the generation of ROS must be kept under control [66]. The major ROS production sources in the vasculature are four pathways including mitochondrial electron transport chain, NADPH oxidases, xanthine oxidase, and endothelial nitric oxide synthase (eNOS) [66]. ROS plays a role in atherosclerosis in various fashions. A major mechanism is lipid oxidation in which accumulated LDL in arterial wall undergoes oxidative modification through ROS produced by NADPH oxidase or uncoupled eNOS [66, 67]. This pathway generates oxidized LDL which is a pro‐atherogenic molecule and causes impairment of NO generation and induction of leukocyte adhesion expression [66, 67]. Another mechanism is the promotion of endothelial dysfunction through induction of NFκB and subsequent promotion of VCAM‐1, ICAM‐1, E‐selectin, and cytokines such as TNF‐α [66]. A noteworthy mechanism is upregulation of oxidized low‐density lipoprotein receptor‐1 (LOX‐1) by oxidized LDL and as a consequence, apoptosis and inflammation are induced concurrent with downregulation of eNOS [66]. Other suggested mechanisms of participation of ROS in the progression of atherosclerosis are DNA oxidation, inflammation, and matrix metalloproteinase (MMP) expression [66]. Oxidized LDL promotes MMP‐1, MMP‐2, and MMP‐9 production that stimulates atherosclerotic plaque rupture and thrombus formation [67]. l‐Arg plays as the substrate for the enzymatic production of NO in endothelial cells, which possess a principal role in vascular tone and cardiovascular homeostasis [7, 9]. NOS isoforms, iNOS, eNOS, and neuronal (nNOS) are the enzyme that produces NO from l‐Arg [7, 8]. The eNOS and nNOS are constitutively expressed, while cytokines and inflammatory responses induce expression of the iNOS [9]. To generate NOS from l‐Arg, first, the arginine is hydroxylated to Nω ‐hydroxy‐arginine, and then it is oxidized into citrulline and NO, and both steps occur via NOS [9]. The oxygenase domain first receives one electron from the reductase domain to form the key ferrous heme intermediate for subsequent oxygen attack, and the second reducing equivalent needed for it to transform further to the peroxy ferric heme comes from a BH4 cofactor adjacent to the heme [68]. BH4 stabilizes the structure of the NOS, enhances the affinity of l‐Arg to NOS, and supports the destabilization of the oxyferrous complex [68]. l‐Arg can modulate the secretion of cytokines and growth factors by myofibroblasts through the arginase pathway, influencing vascular remodeling and atherosclerosis plaque stability [69]. Additionally, l‐Arg affects NOS/arginase balance and subsequently affects antigen‐presenting cells‐derived extracellular vesicles, which can propagate signals that influence atherosclerosis plaque progression [70].

NO is a potent endogenous vasodilator and concurrently possesses anti‐inflammatory, antiproliferative, and anti‐thrombotic properties, thus, it is considered a vasoprotective agent [71]. Low amounts of NO are associated with vascular complications such as hypertension, hypercholesterolemia, diabetes, and atherosclerosis, while large amounts of it react with superoxide onion and involve in pro‐inflammatory reactions and tissue damage [68, 71]. Previous studies demonstrated that the administration of the l‐Arg could lead to vasodilation in atherosclerosis and hypercholesterolemia through NO production, although some studies did not observe any improvement [71]. A recent study showed that the composition of trehalose and l‐Arg as carrier‐free nanomotor facilitated its direct reaction with ROS to stimulate NO production and mitigate the inflammatory microenvironment within atherosclerotic plaques [72]. The low levels of l‐Arg or BH4 cause uncoupling of the reactions and lead to the production of superoxide and/or hydrogen peroxide rather than NO [68]. The ROS are generated by both eNOS and nNOS mainly by the means of oxygenase domain, but the regulation mechanism in eNOS is a decrease in production due to BH4, and in nNOS is attenuation of superoxide and hydrogen peroxide production by l‐Arg [68].

### 3.2 l‐Arg and Cytokines

Cytokines including interleukins (ILs), tumor necrosis factors (TNFs), and several other entities, are a group of molecules that possess an important role in the development of inflammatory pathways [73, 74]. Cytokines are secreted by various cells such as T cells and endothelial cells as a consequence of presence of stimuli and the increase in generation of inflammatory cytokines is associated with development and advancement of atherosclerosis [73, 74]. These molecules participate in progression of atherosclerosis in various means including activation of endothelial cells, endothelial cell dysfunction, promotion of immune cell migration, induction of growth and proliferation of smooth muscle cells, and most outstandingly, atherosclerotic plaque destabilization, apoptosis, and matrix degradation leading to thrombus establishment [73]. IFN‐γ, TNF‐α, and IL‐1β play a role in apoptosis of macrophages and foam cells and as a result, the lipid core of the plaque is enlarged [74]. IFN‐γ and IL‐18 induce the disruption of the plaque and subsequent thrombus formation [74]. A previous study demonstrated that administration of l‐Arg meaningfully diminished the levels of IL‐2, IL‐6, and IFN‐γ which are considered noteworthy pro‐inflammatory cytokines [75]. An increase in levels of TNF‐α, and IL‐1β in the setting of amino acids malnutrition including l‐Arg was observed and this exacerbates oxidative stress and inflammation [76]. A study demonstrated that administration of l‐Arg led to a major reduction in levels of TNF‐α, IL‐1β, IL‐1α, and IL‐6 which are inflammatory cytokines but did not affect the snit‐inflammatory cytokines including IL‐4 and IL‐10 [76]. Concurrent with direct inhibition of inflammatory cytokine production, promoting NO generation by l‐Arg, reduced leukocyte adhesion in post‐ischemic tissue [76].

## 4 Inhibition of Cellular Signaling

### 4.1 NF‐kB Pathway Signaling

l‐Arg has gained attention for its potential in modulating cellular signaling pathways. Specifically, it has been investigated for its role in inhibiting the nuclear factor kappa B (NF‐κB) and TNF pathway signaling. We delved into the mechanisms by which l‐Arg can effectively inhibit these pathways, highlighting its therapeutic implications for various diseases. First, the NF‐kB signaling pathway has been extensively studied and found to play a crucial role in various inflammatory diseases, including atherosclerosis. Atherosclerosis involves complex processes, and key mediators like adhesion molecules and chemokines are implicated in different stages of the disease, ranging from the initiation of plaque formation to plaque rupture. Notably, the recruitment of leukocytes to the vascular intima is a multistep process heavily reliant on chemokines and adhesion molecules, which are regulated through NF‐kB signaling in endothelial cells. The canonical pathway of NF‐kB activation also controls important factors that influence the thrombotic potential of atherosclerotic plaques, such as tissue factor, MMPs, and inflammatory cytokines. Additionally, studies have demonstrated that JNK‐ATF2 signaling‐induced NF‐kB expression in endothelial cells disrupts blood flow, promoting arterial inflammation and sustaining the vascular inflammatory burden, thereby contributing to the development of atherosclerosis. Activation of NF‐kB in endothelial cells triggers the expression of a wide array of adhesion molecules, which orchestrates the invasion of inflammatory cells into the vascular wall and facilitates the migration of smooth muscle cells, leading to remodeling of the extracellular matrix. This critical involvement of the NF‐kB pathway unites these processes in cells of different origins, ultimately driving the progressive accumulation and proliferation of smooth muscle cells in the intima, which significantly contributes to the progression of atherosclerosis. Moreover, studies have investigated the impact of oxidative modification on NF‐kB, revealing its ability to suppress the expression of the γ‐glutamylcysteine synthetase gene in cells treated with oxidized LDL. Interestingly, the effect of oxidized LDL on NF‐kB activation appears to be dose‐dependent, as lower concentrations of oxidized LDL may actually lead to an increase in the expression of pro‐inflammatory genes, including adhesion molecules [77]. NF‐κB is a transcription factor composed of two subunits that play a crucial role in various cellular processes, including inflammation, the immune response of the host, cell adhesion, signaling for growth, cell proliferation, cell differentiation, and defense against apoptosis [78]. Because the NF‐κB transcription factors are fundamental regulators of immunity and inflammation, their defense responses are bioenergetically costly. This requires the immune system to balance protection against pathogens with the need to maintain metabolic homeostasis. Consequently, the activation of NF‐κB signaling is tightly controlled [79]. However, the improper regulation of NF‐κB activation has been implicated in diseases associated with inflammation, including cancers, autoimmune disorders, and conditions with anti‐inflammatory responses. Furthermore, dysregulation of the immune system can lead to chronic inflammation, contributing to the development of long‐term or systemic inflammatory diseases such as rheumatoid arthritis, inflammatory bowel disease, and psoriasis [80]. l‐Arg has shown promise in inhibiting NF‐κB activation through various mechanisms. First, utilizing the antioxidant properties of l‐Arg, it has been demonstrated that it is capable of reducing the production of ROS, thereby decreasing oxidative stress [81]. This decrease will prevent the activation of a variety of transcription factors, mainly NF‐κB, involved in inflammatory pathways. Hence, the reduction of ROS levels by l‐Arg will contribute to mitigate NF‐κB signaling [82]. Additionally, l‐Arg can modulate NF‐κB activation by influencing NO production. l‐Arg acts as a substrate for NOS to synthesize NO [83]. NO, in turn, directly suppresses the activation and expression of NF‐κB, phosphorylates the inhibitor of NF‐κB kinase, and inhibits the transportation of NF‐κB to the nucleus. Consequently, this suppression of the NF‐κB signaling pathway is responsible for the anti‐inflammatory effect, effectively countering the pro‐inflammatory effects of NF‐κB [84]. Moreover, l‐Arg has been observed to exert control over NF‐κB signaling by influencing the expression of pro‐inflammatory cytokines. l‐Arg possesses the capability to decrease the production of pro‐inflammatory cytokines such as IL‐6 and TNF‐α [85]. These cytokines play fundamental roles in NF‐κB‐mediated inflammation. Therefore, by suppressing their levels, the NF‐κB signaling pathway can be attenuated, leading to a dampened inflammatory response [86].

A recent study discovered that the utilization of l‐Arg has the potential to effectively treat significant chronic inflammatory diseases like atherosclerosis. By employing a combination of medicinal plants and bioactive compounds or metabolites, known for their minimal side effects, the study successfully elevated l‐Arg levels. Consequently, this elevation led to increased NO bioavailability, resulting in the improvement of endothelial dysfunction and ultimately preventing the occurrence of atherosclerosis [87].

### 4.2 TNF Pathway Signaling

Extensive research has shed light on the TNF‐α signaling pathway and its potential implication in atherosclerosis progression. It has been found that TNF‐α plays a role in facilitating the transcytosis of lipoproteins, particularly LDL, across endothelial cells. Notably, experiments conducted on human umbilical vein endothelial cells have shown that TNF‐α significantly enhances the transcytosis of LDL, leading to an increased accumulation of LDL within the vascular walls. As a result, TNF‐α‘s involvement in promoting the transcytosis of LDL across endothelial cells contributes to the retention of lipoproteins in the vascular walls, which could further exacerbate the development of atherosclerosis [88]. The TNF‐α plays a vital role in initiating signal transduction pathways by binding to two distinct receptors. These pathways are involved in a range of physiological and pathological processes, including inflammation, immune response, and cell survival and proliferation. However, excessive activation of TNF‐α signaling leads to chronic inflammation, which can eventually result in the development of various pathological complications, including autoimmune diseases [89]. l‐Arg has demonstrated the ability to control the signaling of TNF pathway. Controlling NOS is one way that l‐Arg affects this process. The NOS‐related pathways are responsible for mediating the respiratory effects of TNF‐α and are at the core of how inflammation affects respiratory function [90]. Additionally, l‐Arg has the ability to impact the generation and function of TNF‐α, a crucial cytokine involved in the TNF pathway. Studies had shown that supplementing with l‐Arg can lower the production of TNF‐α in various cell types and animal models [91]. l‐Arg facilitated the reduction in TNF‐α levels, which assists in mitigating the inflammatory effects mediated by the TNF pathway [91]. Furthermore, l‐Arg has previously been identified for its potential to hinder the activation of NF‐κB, which, in turn, can assist in managing the activation of TNF receptors. NF‐κB genes encode NF‐κB dimers, which are instrumental in governing the transcription of various vital inflammatory proteins, including TNF‐α [92].

l‐Arg has been extensively studied for its diverse mechanisms of inhibiting NF‐κB and TNF‐α, making it a promising treatment option for conditions that require the suppression of these pathways, including atherosclerosis. In one particular study, researchers explored the effects of arctium lappa root extract (ALE), a natural extract enriched with l‐Arg as a key component, to assess its potential in mitigating inflammation and improving endothelial dysfunction [93]. The study revealed that ALE exhibited remarkable anti‐inflammatory properties and effectively ameliorated endothelial dysfunction. These beneficial effects were attributed to the suppression of NF‐κB signaling, which consequently led to a reduction in TNF‐α‐induced monocyte adhesion to the vascular endothelium. By impeding the movement of monocytes into the vascular endothelium triggered by TNF‐α, ALE showcased its ability to thwart the initial stages of atherosclerosis. Moreover, the study demonstrated that ALE had a profound impact on the expression of genes responsible for producing inflammatory cytokines such as IL‐1β, IL‐6, TNF‐α, and monocyte chemoattractant protein‐1 (MCP‐1) [93]. The extract successfully reduced the expression of these pro‐inflammatory genes, further supporting its potential as a valuable functional food choice for alleviating atherosclerosis. These compelling findings contribute to the growing body of evidence suggesting the potential therapeutic value of ALE in combatting atherosclerosis [93].

Lastly, l‐Arg has been shown to inhibit the signaling of two important pathways in the human, NF‐κB and TNF. This inhibition occurs through multiple mechanisms. These findings suggest that l‐Arg could be a promising therapeutic target for diseases that are caused by problems with cellular signaling. However, more research and clinical studies are needed to fully understand the therapeutic potential of l‐Arg in various disease conditions.

## 5 Conclusion

L‐Arg affects atherosclerosis in various mechanisms. In atherosclerosis, eNOS may become uncoupled in endothelial cells, producing ROS rather than NO, resulting in endothelial dysfunction. L‐Arg affects the expression of structural proteins (vimentin and tropomyosin) and cytochrome bc1 in coronary venular endothelial cells, which also had beneficial effects on the cellular redox state and enhanced NO production in endothelial cells. L‐Arg inhibited platelet aggregation through the NO pathway. Studies revealed that dysfunctional vascular endothelium, thinning of endothelial glycocalyx, functional alteration induced by impaired NO bioavailability, and elevated level of von Willebrand factor contribute to platelet activation, which l‐Arg has been shown can exert inhibition effects on those factors and furnish stable hemodynamics. l‐Arg reduced neutrophil and macrophage recruitment and delayed time to carotid artery thrombosis. By generating NO when the platelet NO production and responsiveness are suppressed in hypertension, l‐Arg could prevent the monocyte‐platelet aggregates. Another mechanism that l‐Arg affects atherosclerosis is the inhibition of cellular signaling, including NF‐κB and TNF‐α pathways. l‐Arg can modulate NF‐κB activation by influencing NO production. l‐Arg acts as a substrate for NO production. NO directly suppresses the activation and expression of NF‐κB, phosphorylates the inhibitor of NF‐κB kinase, and inhibits the transportation of NF‐κB to the nucleus. Consequently, this suppression of the NF‐κB signaling pathway is responsible for the anti‐inflammatory effect, effectively countering the pro‐inflammatory effects of NF‐κB. l‐Arg has demonstrated the ability to control the signaling of the TNF pathway. Maintaining NOS is one way that l‐Arg affects this process. The NOS‐related pathways mediate the respiratory effects of TNF‐α and are at the core of how inflammation affects respiratory function. Additionally, l‐Arg has the ability to impact the generation and function of TNF‐α, a crucial cytokine involved in the TNF pathway. Therefore, l‐Arg could be considered an alternative therapeutic option for atherosclerosis.

## Author Contributions

**Ali Mortezaei:** writing – original draft, conceptualization, methodology, writing – review and editing, investigation. **Mohammad Ghorbani:** conceptualization, supervision, validation. **Bardia Hajikarimloo:** writing – original draft. **Omar Sameer:** writing – original draft. **Toba Kazemi:** investigation. **Ebrahim Salavati:** investigation. **Mohsen Hamidpour:** investigation, writing – review and editing. **Mohammad Esmaeil Gheydari:** investigation, writing – review and editing.

## Ethics Statement

The authors have nothing to report.

## Consent

The authors have nothing to report.

## Conflicts of Interest

The authors declare no conflicts of interest.

## Transparency Statement

The lead author Mohammad Ghorbani affirms that this manuscript is an honest, accurate, and transparent account of the study being reported; that no important aspects of the study have been omitted; and that any discrepancies from the study as planned (and, if relevant, registered) have been explained.

## Data Availability

The authors have nothing to report.

## References

[1] S. S. Virani , A. Alonso , E. J. Benjamin , et al., “Heart Disease and Stroke Statistics—2020 Update: A Report From the American Heart Association,” Circulation 141, no. 9 (2020): e139–e596. 31992061 10.1161/CIR.0000000000000757

[2] A. Timmis , P. Vardas , N. Townsend , et al., “European Society of Cardiology: Cardiovascular Disease Statistics 2021,” European Heart Journal 43, no. 8 (2022): 716–799. 35016208 10.1093/eurheartj/ehab892

[3] S. Koton , A. L. C. Schneider , W. D. Rosamond , et al., “Stroke Incidence and Mortality Trends in US Communities, 1987 to 2011,” Journal of the American Medical Association 312, no. 3 (2014): 259–268. 25027141 10.1001/jama.2014.7692

[4] K. Nitz , M. Lacy , and D. Atzler , “Amino Acids and Their Metabolism in Atherosclerosis,” Arteriosclerosis, Thrombosis, and Vascular Biology 39, no. 3 (2019): 319–330. 30650999 10.1161/ATVBAHA.118.311572

[5] J. Borén and K. J. Williams , “The Central Role of Arterial Retention of Cholesterol‐Rich Apolipoprotein‐B‐Containing Lipoproteins in the Pathogenesis of Atherosclerosis: A Triumph of Simplicity,” Current Opinion in Lipidology 27, no. 5 (2016): 473–483. 27472409 10.1097/MOL.0000000000000330

[6] B. A. Ference , H. N. Ginsberg , I. Graham , et al., “Low‐Density Lipoproteins Cause Atherosclerotic Cardiovascular Disease. 1. Evidence From Genetic, Epidemiologic, and Clinical Studies. A Consensus Statement From the European Atherosclerosis Society Consensus Panel,” European Heart Journal 38, no. 32 (2017): 2459–2472. 28444290 10.1093/eurheartj/ehx144 PMC5837225

[7] J. Ramachandran and R. D. Peluffo , “Threshold Levels of Extracellular L‐Arginine That Trigger NOS‐Mediated ROS/RNS Production in Cardiac Ventricular Myocytes,” American Journal of Physiology‐Cell Physiology 312, no. 2 (2017): C144–C154. 27903582 10.1152/ajpcell.00150.2016 PMC5336593

[8] L. Rochette , J. Lorin , M. Zeller , et al., “Nitric Oxide Synthase Inhibition and Oxidative Stress in Cardiovascular Diseases: Possible Therapeutic Targets?,” Pharmacology & Therapeutics 140, no. 3 (2013): 239–257. 23859953 10.1016/j.pharmthera.2013.07.004

[9] J. Gambardella , W. Khondkar , M. B. Morelli , X. Wang , G. Santulli , and V. Trimarco , “Arginine and Endothelial Function,” Biomedicines 8, no. 8 (2020): 277. 32781796 10.3390/biomedicines8080277 PMC7460461

[10] P. M. Ridker , B. M. Everett , T. Thuren , et al., “Antiinflammatory Therapy With Canakinumab for Atherosclerotic Disease,” New England Journal of Medicine 377, no. 12 (2017): 1119–1131. 28845751 10.1056/NEJMoa1707914

[11] Q. Tong , X. Yang , S. R. Ma , X. F. Zhang , Y. Wang , and J. D. Jiang , 2020. Hydroxyurea Regressed Atherosclerosis Plaques in ApoE‐/‐Mice: A Discovery Based on Clinic.

[12] R. Zhang , S. Chen , H. Zhang , et al., “Effects of Methotrexate in a Rabbit Model of in‐Stent Neoatherosclerosis: An Optical Coherence Tomography Study,” Scientific Reports 6, no. 1 (2016): 33657. 27644847 10.1038/srep33657 PMC5028880

[13] H. Li , M. I. Cybulsky , M. A. Gimbrone , and P. Libby , “An Atherogenic Diet Rapidly Induces VCAM‐1, a Cytokine‐Regulatable Mononuclear Leukocyte Adhesion Molecule, in Rabbit Aortic Endothelium,” Arteriosclerosis and Thrombosis: A Journal of Vascular Biology 13, no. 2 (1993): 197–204. 7678986 10.1161/01.atv.13.2.197

[14] M. I. Cybulsky and M. A. Gimbrone , “Endothelial Expression of a Mononuclear Leukocyte Adhesion Molecule During Atherogenesis,” Science 251, no. 4995 (1991): 788–791. 1990440 10.1126/science.1990440

[15] K. Hu , Y. Li , Z. Ke , et al., “History, Progress and Future Challenges of Artificial Blood Vessels: A Narrative Review,” Biomater Transl 3, no. 1 (2022 March 28): 81–98. 35837341 10.12336/biomatertransl.2022.01.008 PMC9255792

[16] S. Mehran , S. Ghodratizadeh , A. Zolfi‐Gol , et al., “Titanium Dioxide Nanoparticle and Cardiovascular Diseases: A Critical Review of the Literature and Possible Underlying Mechanisms,” Nano Biomedicine and Engineering 14 (2022): 323–336.

[17] N. J. Alp and K. M. Channon , “Regulation of Endothelial Nitric Oxide Synthase by Tetrahydrobiopterin in Vascular Disease,” Arteriosclerosis, Thrombosis, and Vascular Biology 24, no. 3 (2004): 413–420. 14656731 10.1161/01.ATV.0000110785.96039.f6

[18] H. Li and U. Förstermann , “Uncoupling of Endothelial NO Synthase in Atherosclerosis and Vascular Disease,” Current Opinion in Pharmacology 13, no. 2 (2013): 161–167. 23395155 10.1016/j.coph.2013.01.006

[19] P. J. Kuhlencordt , J. Chen , F. Han , J. Astern , and P. L. Huang , “Genetic Deficiency of Inducible Nitric Oxide Synthase Reduces Atherosclerosis and Lowers Plasma Lipid Peroxides in Apolipoprotein E–Knockout Mice,” Circulation 103, no. 25 (2001): 3099–3104. 11425775 10.1161/01.cir.103.25.3099

[20] W. Shi , X. Wang , D. M. Shih , V. E. Laubach , M. Navab , and A. J. Lusis , “Paradoxical Reduction of Fatty Streak Formation in Mice Lacking Endothelial Nitric Oxide Synthase,” Circulation 105, no. 17 (2002): 2078–2082. 11980688 10.1161/01.cir.0000015853.59427.32

[21] J. J. Chiu and S. Chien , “Effects of Disturbed Flow on Vascular Endothelium: Pathophysiological Basis and Clinical Perspectives,” Physiological Reviews 91, no. 1 (2011 January): 327–387. 21248169 10.1152/physrev.00047.2009 PMC3844671

[22] Y. Lin , R. Lin , H. B. Lin , and S. Shen , “Nanomedicine‐Based Drug Delivery Strategies for the Treatment of Atherosclerosis,” Medicine in Drug Discovery 22 (2024 Jun 1): 100189.

[23] S. Wang , Y. Wang , X. Lai , et al., “Minimalist Nanocomplex With Dual Regulation of Endothelial Function and Inflammation for Targeted Therapy of Inflammatory Vascular Diseases,” ACS Nano 17, no. 3 (2023 February 14): 2761–2781. 36719043 10.1021/acsnano.2c11058

[24] M. Yáñez‐Mó , P. R. M. Siljander , Z. Andreu , et al., “Biological Properties of Extracellular Vesicles and Their Physiological Functions,” Journal of Extracellular Vesicles 4 (2015): 27066. 25979354 10.3402/jev.v4.27066 PMC4433489

[25] X. Lei , C. Feng , C. Liu , et al., “Regulation of Protein Expression by L‐Arginine in Endothelial Cells,” Frontiers in Bioscience (Scholar Edition) 3, no. 2 (2011): 655–661. 21196403 10.2741/s178

[26] S. H. Javanmard , Y. Gheisari , M. Soleimani , M. Nematbakhsh , and A. Monajemi , “Effect of L‐Arginine on Circulating Endothelial Progenitor Cells in Hypercholesterolemic Rabbits,” International Journal of Cardiology 143, no. 2 (2010): 213–216. 19167766 10.1016/j.ijcard.2008.11.203

[27] P. Theofilis , M. Sagris , A. S. Antonopoulos , E. Oikonomou , C. Tsioufis , and D. Tousoulis , “Inflammatory Mediators of Platelet Activation: Focus on Atherosclerosis and COVID‐19,” International Journal of Molecular Sciences 22, no. 20 (2021): 11170. 34681830 10.3390/ijms222011170 PMC8539848

[28] M. W. Radomski , R. M. Palmer , and S. Moncada , “Glucocorticoids Inhibit the Expression of an Inducible, but Not the Constitutive, Nitric Oxide Synthase in Vascular Endothelial Cells,” Proceedings of the National Academy of Sciences 87, no. 24 (1990): 10043–10047. 10.1073/pnas.87.24.10043 PMC55311 1702214

[29] C. J. Forsyth , M. R. Adams , W. Jessup , J. Robinson , and D. S. Celermajer , 1995 “Oral L‐Arginine Inhibits Platelet Aggregation but Does not Enhance Endothelium‐Dependent Dilation in Healthy Young Men” *Journal of the American College of Cardiology* 26, no. 4 (1995): 1054–1061, https://elibrary.cclhd.health.nsw.gov.au/cclhdjspui/handle/1/1367. 10.1016/0735-1097(95)00257-9 7560599

[30] P. Canzano , M. Brambilla , B. Porro , et al., “Platelet and Endothelial Activation as Potential Mechanisms Behind the Thrombotic Complications of COVID‐19 Patients,” JACC: Basic to Translational Science 6, no. 3 (2021): 202–218. 33649738 10.1016/j.jacbts.2020.12.009 PMC7904280

[31] A. Sacchi , G. Grassi , S. Notari , et al., “Expansion of Myeloid Derived Suppressor Cells Contributes to Platelet Activation by L‐Arginine Deprivation During SARS‐CoV‐2 Infection,” Cells 10, no. 8 (2021 August 17): 2111. 34440879 10.3390/cells10082111 PMC8391818

[32] A. Sacchi , G. Grassi , V. Bordoni , et al., “Early Expansion of Myeloid‐Derived Suppressor Cells Inhibits SARS‐CoV‐2 Specific T‐Cell Response and May Predict Fatal COVID‐19 Outcome,” *Cell Death & Disease*, 11, no. 921 (2020), https://www.nature.com/articles/s41419-020-03125-1. 10.1038/s41419-020-03125-1 PMC7590570 33110074

[33] K. Ley , C. Laudanna , M. I. Cybulsky , and S. Nourshargh , “Getting to the Site of Inflammation: The Leukocyte Adhesion Cascade Updated,” Nature Reviews Immunology 7, no. 9 (2007 September): 678–689. 10.1038/nri2156 17717539

[34] E. Checkouri , V. Blanchard , and O. Meilhac , “Macrophages in Atherosclerosis, First or Second Row Players?,” Biomedicines 9, no. 9 (2021 September): 1214. 34572399 10.3390/biomedicines9091214 PMC8465019

[35] M. Munder , “Suppression of T‐Cell Functions by Human Granulocyte Arginase,” Blood 108, no. 5 (2006): 1627–1634. 16709924 10.1182/blood-2006-11-010389

[36] J. Oberlies , C. Watzl , T. Giese , et al., “Regulation of NK Cell Function by Human Granulocyte Arginase,” Journal of Immunology 182, no. 9 (2009): 5259–5267. 10.4049/jimmunol.0803523 19380772

[37] H. Sato , H. Sato , Z. Q. Zhao , and J. Vinten‐Johansen , “L‐Arginine Inhibits Neutrophil Adherence and Coronary Artery Dysfunction,” Cardiovascular Research 31, no. 1 (1996): 63–72. 8849590

[38] M. R. Adams , W. Jessup , D. Hailstones , and D. S. Celermajer , “L‐Arginine Reduces Human Monocyte Adhesion to Vascular Endothelium and Endothelial Expression of Cell Adhesion Molecules,” Circulation 95, no. 3 (1997): 662–668. 9024155 10.1161/01.cir.95.3.662

[39] R. Fitterer , T. Lajqi , S. A. Kranig , et al., “L‐Arginine Modulates Neonatal Leukocyte Recruitment in a Gestational Age‐Dependent Manner,” Journal of Clinical Medicine 9, no. 9 (2020): 2772. 32867030 10.3390/jcm9092772 PMC7563285

[40] M. Rattazzi , M. Donato , E. Bertacco , et al., “L‐Arginine Prevents Inflammatory and Pro‐Calcific Differentiation of Interstitial Aortic Valve Cells,” Atherosclerosis 298 (2020): 27–35, https://www.atherosclerosis-journal.com/article/S0021-9150(20)30115-5/fulltext. 32169720 10.1016/j.atherosclerosis.2020.02.024

[41] J. S. Knight , W. Luo , A. A. O'Dell , et al., “Peptidylarginine Deiminase Inhibition Reduces Vascular Damage and Modulates Innate Immune Responses in Murine Models of Atherosclerosis,” Circulation Research 114, no. 6 (2014): 947–956. 24425713 10.1161/CIRCRESAHA.114.303312 PMC4185401

[42] A. E. Vendrov , K. C. Vendrov , A. Smith , et al., “NOX4 NADPH Oxidase‐Dependent Mitochondrial Oxidative Stress in Aging‐Associated Cardiovascular Disease,” Antioxidants & Redox Signaling 23, no. 18 (2015 December 20): 1389–1409. 26054376 10.1089/ars.2014.6221 PMC4692134

[43] P. H. Becker , P. Thérond , and P. Gaignard , “Targeting Mitochondrial Function in Macrophages: A Novel Treatment Strategy for Atherosclerotic Cardiovascular Disease?,” Pharmacology & Therapeutics 247 (2023 July 1): 108441. 37201736 10.1016/j.pharmthera.2023.108441

[44] G. G. Dorighello , B. A. Paim , S. F. Kiihl , et al., “Correlation Between Mitochondrial Reactive Oxygen and Severity of Atherosclerosis,” Oxidative Medicine and Cellular Longevity 2016 (2016): 7843685. 26635912 10.1155/2016/7843685 PMC4655284

[45] E. P. K. Yu , J. Reinhold , H. Yu , et al., “Mitochondrial Respiration Is Reduced in Atherosclerosis, Promoting Necrotic Core Formation and Reducing Relative Fibrous Cap Thickness,” Arteriosclerosis, Thrombosis, and Vascular Biology 37, no. 12 (2017 Dec): 2322–2332. 28970293 10.1161/ATVBAHA.117.310042 PMC5701734

[46] B. H. Koo , B. G. Yi , W. K. Wang , et al., “Arginase Inhibition Suppresses Native Low‐Density Lipoprotein‐Stimulated Vascular Smooth Muscle Cell Proliferation by NADPH Oxidase Inactivation,” Yonsei Medical Journal 59, no. 3 (2018): 366–375. 29611398 10.3349/ymj.2018.59.3.366 PMC5889988

[47] A. Sachinidis , T. Mengden , R. Locher , C. Brunner , and W. Vetter , “Novel Cellular Activities for Low Density Lipoprotein in Vascular Smooth Muscle Cells,” Hypertension 15, no. 6 pt 2 (1990): 704–711. 2351425 10.1161/01.hyp.15.6.704

[48] R. Locher , R. P. Brandes , W. Vetter , and M. Barton , “Native LDL Induces Proliferation of Human Vascular Smooth Muscle Cells Via Redox‐Mediated Activation of ERK 1/2 Mitogen‐Activated Protein Kinases,” Hypertension 39, no. 2 (2002): 645–650. 11882624 10.1161/hy0202.103473

[49] A. M. Wilson , R. Harada , N. Nair , N. Balasubramanian , and J. P. Cooke , “L‐Arginine Supplementation in Peripheral Arterial Disease: No Benefit and Possible Harm,” Circulation 116, no. 2 (2007): 188–195. 17592080 10.1161/CIRCULATIONAHA.106.683656

[50] J. G. McCormack and R. M. Denton , “Mitochondrial Ca^2+^ Transport and the Role of Intramitochondrial Ca^2^ ^+^ in the Regulation of Energy Metabolism,” Developmental Neuroscience 15, no. 3–5 (1993): 165–173. 7805568 10.1159/000111332

[51] R. S. Balaban , “Cardiac Energy Metabolism Homeostasis: Role of Cytosolic Calcium,” Journal of Molecular and Cellular Cardiology 34, no. 10 (2002): 1259–1271. 12392982 10.1006/jmcc.2002.2082

[52] Koo B. H. Hyeock , D. Hong , H. D. Hong , et al., “Arginase II Activity Regulates Cytosolic Ca^2+^ Level in a p32‐Dependent Manner That Contributes to Ca^2+^‐Dependent Vasoconstriction in Native Low‐Density Lipoprotein‐Stimulated Vascular Smooth Muscle Cells,” Experimental & Molecular Medicine 51, no. 6 (2019): 1–12. 10.1038/s12276-019-0262-y PMC6545325 31155612

[53] P. Singh , J. Sun , M. Cavalera , et al., “Dysregulation of MMP2‐Dependent TGF‐ß2 Activation Impairs Fibrous Cap Formation in Type 2 Diabetes‐Associated Atherosclerosis,” Nature Communications 15, no. 1 (2024 December 9): 10464. 10.1038/s41467-024-50753-8 PMC11628557 39653743

[54] T. Lisman , “Platelet–Neutrophil Interactions as Drivers of Inflammatory and Thrombotic Disease,” Cell and Tissue Research 371, no. 3 (2018): 567–576. 29178039 10.1007/s00441-017-2727-4 PMC5820397

[55] X. R. Xu , D. Zhang , B. E. Oswald , et al., “Platelets Are Versatile Cells: New Discoveries in Hemostasis, Thrombosis, Immune Responses, Tumor Metastasis and Beyond,” Critical Reviews in Clinical Laboratory Sciences 53, no. 6 (2016): 409–430. 27282765 10.1080/10408363.2016.1200008

[56] M. Ghorbani , D. Bashash , M. E. Gheydari , et al., “Platelet–Leukocyte Aggregate and Interleukin‐6: An Emerging Perspective on a New Diagnostic and Therapeutic Clue for Acute Coronary Syndrome, a Case–Control Study,” Health Science Reports 7, no. 12 (2024): e70209. 39669190 10.1002/hsr2.70209 PMC11635124

[57] H. Shahraki , M. E. Gheydari , M. H. Mohammadi , et al., “Mac‐1 Alongside Platelet‐Monocyte Aggregates as Potential Markers in Acute Coronary Syndrome: A Case‐Control Study,” Cell Journal (Yakhteh) 26, no. 7 (2024 September 11): 454–464. 10.22074/cellj.2024.2024525.1527 39290123

[58] A. Gisterå and G. K. Hansson , “The Immunology of Atherosclerosis,” Nature Reviews Nephrology 13, no. 6 (2017 June): 368–380. 28392564 10.1038/nrneph.2017.51

[59] A. D. Shah , S. Denaxas , O. Nicholas , A. D. Hingorani , and H. Hemingway , “Neutrophil Counts and Initial Presentation of 12 Cardiovascular Diseases,” Journal of the American College of Cardiology 69, no. 9 (2017 March 7): 1160–1169. 28254179 10.1016/j.jacc.2016.12.022 PMC5332591

[60] B. D. Horne , J. L. Anderson , J. M. John , et al., “Which White Blood Cell Subtypes Predict Increased Cardiovascular Risk?,” Journal of the American College of Cardiology 45, no. 10 (2005 May 17): 1638–1643. 15893180 10.1016/j.jacc.2005.02.054

[61] K. Distelmaier , M. P. Winter , F. Dragschitz , et al., “Prognostic Value of Culprit Site Neutrophils in Acute Coronary Syndrome,” European Journal of Clinical Investigation 44, no. 3 (2014): 257–265. 24720533 10.1111/eci.12228

[62] I. C. Moschonas and A. D. Tselepis , “The Pathway of Neutrophil Extracellular Traps Towards Atherosclerosis and Thrombosis,” Atherosclerosis 288 (2019 September): 9–16. 31280097 10.1016/j.atherosclerosis.2019.06.919

[63] A. Warnatsch , M. Ioannou , Q. Wang , and V. Papayannopoulos , “Neutrophil Extracellular Traps License Macrophages for Cytokine Production in Atherosclerosis,” Science 349, no. 6245 (2015 July 17): 316–320. 26185250 10.1126/science.aaa8064 PMC4854322

[64] Y. Döring , P. Libby , and O. Soehnlein , “Neutrophil Extracellular Traps Participate in Cardiovascular Diseases: Recent Experimental and Clinical Insights,” Circulation Research 126, no. 9 (2020): 1228–1241. 32324499 10.1161/CIRCRESAHA.120.315931 PMC7185047

[65] E. Gkaliagkousi , V. Corrigall , S. Becker , et al., “Decreased Platelet Nitric Oxide Contributes to Increased Circulating Monocyte‐Platelet Aggregates in Hypertension,” European Heart Journal 30, no. 24 (2009): 3048–3054. 19687162 10.1093/eurheartj/ehp330

[66] M. Batty , M. R. Bennett , and E. Yu , “The Role of Oxidative Stress in Atherosclerosis,” Cells 11, no. 23 (2022): 3843. 36497101 10.3390/cells11233843 PMC9735601

[67] A. J. Kattoor , N. V. K. Pothineni , D. Palagiri , and J. L. Mehta , “Oxidative Stress in Atherosclerosis,” Current Atherosclerosis Reports 19, no. 11 (2017): 42. 28921056 10.1007/s11883-017-0678-6

[68] V. Berka , L. H. Wang , and A. L. Tsai , “Oxygen‐Induced Radical Intermediates in the nNos Oxygenase Domain Regulated by L‐Arginine, Tetrahydrobiopterin, and Thiol,” Biochemistry 47, no. 1 (2008): 405–420. 18052254 10.1021/bi701677r

[69] A. Mahdi , O. Kövamees , and J. Pernow , “Improvement in Endothelial Function in Cardiovascular Disease—Is Arginase the Target?,” International Journal of Cardiology 301 (2020 February 15): 207–214. 31785959 10.1016/j.ijcard.2019.11.004

[70] A. Mantovani , S. K. Biswas , M. R. Galdiero , A. Sica , and M. Locati , “Macrophage Plasticity and Polarization in Tissue Repair and Remodelling,” Journal of Pathology 229, no. 2 (2013 January): 176–185. 23096265 10.1002/path.4133

[71] J. Lorin , M. Zeller , J. C. Guilland , Y. Cottin , C. Vergely , and L. Rochette , “Arginine and Nitric Oxide Synthase: Regulatory Mechanisms and Cardiovascular Aspects,” Molecular Nutrition & Food Research 58, no. 1 (2014): 101–116. 23740826 10.1002/mnfr.201300033

[72] Z. Wu , M. Zhou , X. Tang , et al., “Carrier‐Free Trehalose‐Based Nanomotors Targeting Macrophages in Inflammatory Plaque for Treatment of Atherosclerosis,” ACS Nano 16, no. 3 (2022 March 22): 3808–3820. 35199998 10.1021/acsnano.1c08391

[73] A. R. Fatkhullina , I. O. Peshkova , and E. K. Koltsova , “The Role of Cytokines in the Development of Atherosclerosis,” Biochemistry (Moscow) 81 (2016): 1358–1370. 27914461 10.1134/S0006297916110134 PMC5471837

[74] D. P. Ramji and T. S. Davies , “Cytokines in Atherosclerosis: Key Players in All Stages of Disease and Promising Therapeutic Targets,” Cytokine & Growth Factor Reviews 26, no. 6 (2015): 673–685. 26005197 10.1016/j.cytogfr.2015.04.003 PMC4671520

[75] V. Trimarco , R. Izzo , A. Lombardi , A. Coppola , G. Fiorentino , and G. Santulli , “Beneficial Effects of L‐Arginine in Patients Hospitalized for COVID‐19: New Insights From a Randomized Clinical Trial,” Pharmacological Research 191 (2023): 106702. 36804278 10.1016/j.phrs.2023.106702 PMC9928676

[76] A. A. Korish , “Multiple Antioxidants and L‐Arginine Modulate Inflammation and Dyslipidemia in Chronic Renal Failure Rats,” Renal Failure 32, no. 2 (2010): 203–213. 20199183 10.3109/08860221003592820

[77] B. Pamukcu , G. Y. H. Lip , and E. Shantsila , “The Nuclear Factor–Kappa B Pathway in Atherosclerosis: A Potential Therapeutic Target for Atherothrombotic Vascular Disease,” Thrombosis Research 128, no. 2 (2011): 117–123. 21636112 10.1016/j.thromres.2011.03.025

[78] K. S. Alharbi , N. K. Fuloria , S. Fuloria , et al., “Nuclear Factor‐Kappa B and Its Role in Inflammatory Lung Disease,” Chemico‐Biological Interactions 345 (2021): 109568. 34181887 10.1016/j.cbi.2021.109568

[79] D. Capece , D. Verzella , I. Flati , P. Arboretto , J. Cornice , and G. Franzoso , “NF‐κB: Blending Metabolism, Immunity, and Inflammation,” Trends in Immunology 43, no. 9 (2022): 757–775. 35965153 10.1016/j.it.2022.07.004

[80] H. Yu , L. Lin , Z. Zhang , H. Zhang , and H. Hu , “Targeting NF‐κB Pathway for the Therapy of Diseases: Mechanism and Clinical Study,” Signal Transduction and Targeted Therapy 5, no. 1 (2020): 209. 32958760 10.1038/s41392-020-00312-6 PMC7506548

[81] M. M. Sayed , N. M. Abd el‐Rady , W. M. S. Gomaa , A. Hosny , and A. M. S. Gomaa , “Antioxidant, Antiapoptotic, and Antifibrotic Abilities of L‐Arginine Ameliorate the Testicular Dysfunction in Diabetic Rats,” Tissue and Cell 82 (2023): 102036. 36841127 10.1016/j.tice.2023.102036

[82] O. J. Sul and S. W. Ra , “Quercetin Prevents LPS‐Induced Oxidative Stress and Inflammation by Modulating NOX2/ROS/NF‐kB in Lung Epithelial Cells,” Molecules 26, no. 22 (2021): 6949. 34834040 10.3390/molecules26226949 PMC8625571

[83] D. Khalaf , M. Krüger , M. Wehland , M. Infanger , and D. Grimm , “The Effects of Oral L‐Arginine and L‐Citrulline Supplementation on Blood Pressure,” Nutrients 11, no. 7 (2019): 1679. 31336573 10.3390/nu11071679 PMC6683098

[84] Q. X. Kuang , Y. Luo , L. R. Lei , et al., “Hydroanthraquinones From Nigrospora Sphaerica and Their Anti‐Inflammatory Activity Uncovered by Transcriptome Analysis,” Journal of Natural Products 85, no. 6 (2022): 1474–1485. 35696541 10.1021/acs.jnatprod.1c01141

[85] J. M. Pérez de la Lastra , C. M. Curieses Andrés , C. Andrés Juan , F. J. Plou , and E. Pérez‐Lebeña , “Hydroxytyrosol and Arginine as Antioxidant, Anti‐Inflammatory and Immunostimulant Dietary Supplements for COVID‐19 and Long COVID,” Foods 12, no. 10 (2023): 1937. 37238755 10.3390/foods12101937 PMC10217518

[86] J. L. Pang , J. W. Wang , P. Y. Hu , J. S. Jiang , and C. Yu , “HOTAIR Alleviates Ox‐LDL‐Induced Inflammatory Response in Raw264. 7 Cells via Inhibiting NF‐κB Pathway,” European Review for Medical and Pharmacological Sciences 22, no. 20 (2018): 6991–6998. 30402866 10.26355/eurrev_201810_16170

[87] K. Malekmohammad , R. D. E. Sewell , and M. Rafieian‐Kopaei , “Mechanisms of Medicinal Plant Activity on Nitric Oxide (NO) Bioavailability as Prospective Treatments for Atherosclerosis,” Current Pharmaceutical Design 26, no. 22 (2020): 2591–2601. 32188375 10.2174/1381612826666200318152049

[88] Y. Zhang , X. Yang , F. Bian , et al., “TNF‐α Promotes Early Atherosclerosis by Increasing Transcytosis of LDL Across Endothelial Cells: Crosstalk Between NF‐κB and PPAR‐γ,” Journal of Molecular and Cellular Cardiology 72 (2014): 85–94. 24594319 10.1016/j.yjmcc.2014.02.012

[89] D. Jang , A. H. Lee , H. Y. Shin , et al., “The Role of Tumor Necrosis Factor Alpha (TNF‐α) in Autoimmune Disease and Current TNF‐α Inhibitors in Therapeutics,” International Journal of Molecular Sciences 22, no. 5 (2021): 2719. 33800290 10.3390/ijms22052719 PMC7962638

[90] N. P. Aleksandrova , A. A. Klinnikova , and G. A. Danilova , “Cyclooxygenase and Nitric Oxide Synthase Pathways Mediate the Respiratory Effects of TNF‐α in Rats,” Respiratory Physiology & Neurobiology 284 (2021): 103567. 33161117 10.1016/j.resp.2020.103567

[91] V. S. Chetla , S. Bommu , N. A. Laxmi , K. Putty , K. K. Reddy , and K. K. Bharani , “Comparative Evaluation of the Effect of L‐Arginine and L‐Homoarginine Supplementation on Reproductive Physiology in Ewes,” Research in Veterinary Science 149 (2022): 159–171. 35841692 10.1016/j.rvsc.2022.06.022

[92] L. Acar , N. Atalan , E. H. Karagedik , and A. Ergen , “Tumour Necrosis Factor‐Alpha and Nuclear Factor‐Kappa B Gene Variants in Sepsis,” Balkan Medical Journal 35, no. 1 (2018): 30–35. 28840846 10.4274/balkanmedj.2017.0246 PMC5820445

[93] J. Lee , S. J. Ha , J. Park , et al., “Arctium Lappa Root Extract Containing L‐Arginine Prevents TNF‐α‐Induced Early Atherosclerosis in Vitro and in Vivo,” Nutrition Research 77 (2020): 85–96. 32388084 10.1016/j.nutres.2020.03.003

